# Regional longitudinal bi-ventricular function in pulmonary hypertension: single heart-beat assessment of strain by fast-senc imaging

**DOI:** 10.1186/1532-429X-13-S1-P279

**Published:** 2011-02-02

**Authors:** Monda L Shehata, Tamer A Basha, Ahmed A Harouni, Jan Skrok, Sukhminder Singh, Stephen Mathai, Joao AC Lima, Nael F Osman, David A Bluemke, Paul M Hassoun, Jens Vogel-Claussen

**Affiliations:** 1 Johns Hokins University, Baltimore, MD, USA; 2 National Institutes of Health, Bethesda, MD, USA

## Purpose

Right ventricular (RV) function is the most important determinant of survival in patients with pulmonary hypertension (PH), thus monitoring of RV function is critical. However, RV regional function assessment is challenging using current MR tagging techniques. Thus, the purpose was to evaluate regional longitudinal ventricular deformation (E_LL_) acquired by free breathing single heart-beat fast strain encoded imaging (fast-SENC) in relation to global ventricular dysfunction markers and pulmonary hemodynamics in PH patients.

## Materials and methods

48 subjects [35 PH patients (mean pulmonary arterial pressure mPAP = 40.2±11.8 mmHg) and 13 age and gender matched controls] were examined using short axis fast-SENC MRI and cine imaging. All patients underwent right heart catheterization (RHC). Segmental (15 RV and 16 LV segments), slice (basal, mid and apical), as well as mean (average of segments) peak systolic E_LL_ were quantified for both ventricles and correlated with global function and RHC indices. Patients were stratified into 3 groups based on RV ejection fraction (RVEF).

## Results

**Figure 1:**
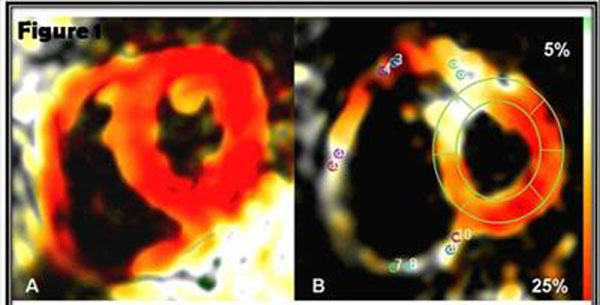
(A) Basal short axis fast-SENC peak systolic image in a 53 year old healthy female subject demonstrating normal longitudinal shortening (E_LL_) (red color). (B) Basal short axis fast-SENC peak systolic image in a 47 year old female PH patient (mPAP = 51 mmHg). Note regionally reduced RV E_LL_ and LV antero-septal E_LL_ (white color). Probe points correspond to regions of interest where strain was measured. A mesh was used to measure LV E_LL_ at six basal, six mid and four apical LV segments respectively.

**Figure 2:**
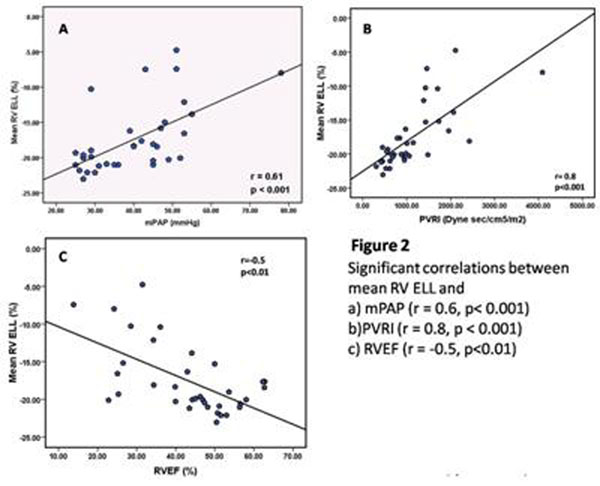


PH patients demonstrated reduced E_LL_ at all RV levels compared to controls (Figure 1). On regional analysis, reduced E_LL_ was noted in all segments (p<0.05) except the inferior and inferior septal insertion at all levels (Table 1). Reduced mean RV E_LL_ correlated with elevated mPAP (r=0.6, p<0.001), pulmonary vascular resistance index (PVRI, r=0.8, p<0.001) as well as reduced RV systolic function parameters (p<0.05 for all) (Figure 2). In the LV, reduced E_LL_ was mainly noted at the basal anterior (-16.7 vs. -20.5, p=0.03) and antero-septal regions (-12.8 vs. -18.2, p<0.01). Reduced LV antero-septal E_LL_ correlated with increased mPAP (r=0.5, p<0.01), increased septal eccentricity index (r=0.5, p<0.01) and reduced RV systolic function (p<0.05 for all). On multiple linear regression including mPAP, RV end-diastolic volume index and RV mass index as covariates, mPAP was an independent predictor of reduced mean RV E_LL_ (β=0.19, p<0.01). In turn, reduced mean RV E_LL_ was the main predictor of reduced RVEF in PH patients (β=-1.2, p=0.03) in a model including mPAP, mean RV E_LL_ and RV mass index. In PH patients with maintained global RV function, regional E_LL_ was reduced at the basal and mid anterior septal insertions as well as mid anterior RV segments (p<0.05 for all).

**Table 1:** Right ventricular longitudinal (ELL) strain in PH patients and controls

E_LL_ (%)	PH 25% - 75% (N=35)	Control 25% - 75% (N =13)	P value
Mean Ventricular Strain	-19.3 (-20.5 - -15.3)	-21.3 (-21.9 - -20.2)	0.001
Slices			
Basal	-19.1 (-21.6 - -14.9)	-21.7 (-22.0 - -20.2)	0.008
Mid	-19.3 (-20.3 - -12.6)	-21.8 (-22.7 - -19.1)	0.002
Apical	-18.1 (-21.8 - -15.7)	-20.9 (-22.3 - -19.8)	0.02
Segments			
Basal Anterior Septal Insertion	-14.1 (-18.8 - -12.5)	-20.2 (-23.1 - -17.1)	<0.001
Basal Anterior	-21.2 (-24.1 - -15.1)	-24.0 (-25.1 - -21.8)	0.02
Basal Lateral	-21.0 (-25.1 - -17.9)	-23.8 (-26.2 - -22.5)	0.03
Basal Inferior	-18.3 (-21.6 - -15.1)	-20.2 (-23.6 - -18.6)	0.09
Basal Inferior Septal Insertion	-19.9 (-21.6 - -14.1)	-19.1 (-21.7 - -16.3)	0.88
Mid Anterior Septal Insertion	-15.1 (-18.1 - -9.6)	-20.8 (-22.7 - -17.7)	<0.001
Mid Anterior	-16.3 (-19.1 - -12.5)	-22.1 (-25.1 - -21.3)	<0.001
Mid Lateral	-22.5 (-23.6 - -15.0)	-24.9 (-25.9 - -22.9)	0.005
Mid Inferior	-20.1 (-23.3 - -14.1)	-20.1 (-24.6 - -18.7)	0.16
Mid Inferior Septal Insetion	-16.9 (-21.2 - -11.1)	-18.7 (-20.1 - -16.1)	0.49
Apical Anterior Septal Insertion	-16.6 (-20.7 - -12.3)	-20.4 (-22.3 - -19.0)	0.02
Apical Anterior	-18.4 (-21.2 - -14.9)	-21.9 (-23.8 - -18.8)	0.01
Apical Lateral	-20.4 (-24.4 - -15.4)	-24.1 (-25.7 - - 22.1)	0.0009
Apical Inferior	-18.3 (-24.1 - -13.1)	-22.2 (-24.6 - -19.7)	0.08
Apical Inferior Septal Insertion	-18.1 (-21.8 - -12.8)	-17.3 (-19.5 - -15.6)	0.84

## Conclusion

In PH patients, reduced RV E_LL_ measured by fast-SENC is associated with increased afterload and correlates with biventricular global dysfunction. RV strain analysis using fast-SENC can detect subclinical regional dysfunction in absence of global RV functional compromise.

